# Rational Design of a Chimpanzee Adenoviral-Vector Vaccine Against Yellow Fever Through the Modification of Antigen Transmembrane Domains

**DOI:** 10.3390/vaccines14030273

**Published:** 2026-03-20

**Authors:** Marta Ulaszewska, Ji Ma, Susan J. Morris, Sophie M. Jegouic Goodall, Winnie Kerstens, Hendrik Jan Thibaut, Lotte Coelmont, Kai Dallmeier, Sarah C. Gilbert, Barbara Dema

**Affiliations:** 1 Pandemic Sciences Institute, Nuffield Department of Medicine, University of Oxford, Oxford OX3 7TY, UK; 2 Virology, Antiviral Drug & Vaccine Research Group, Laboratory of Molecular Vaccinology and Vaccine Discovery (MVVD), KU Leuven Department of Microbiology, Immunology and Transplantation, Rega Institute, 3000 Leuven, Belgium; 3 Virology, Antiviral Drug & Vaccine Research Group, Translational Platform for Virus, Vaccine and Cancer Research (TPVC), KU Leuven Department of Microbiology, Immunology and Transplantation, Rega Institute, 3000 Leuven, Belgium; 4 CAMS Oxford Institute, Chinese Academy of Medical Sciences & Peking Union Medical College, University of Oxford, Oxford OX3 7BN, UK

**Keywords:** yellow fever, ChAdOx1, flavivirus, vaccine, adenoviral vector, immunogenicity, efficacy

## Abstract

**Background/Objectives**: Chimpanzee adenoviral-vectored vaccines have proven to be both safe and effective, with a manufacturing and distribution pipeline capable of rapid global supply, as demonstrated during the COVID-19 pandemic. Yellow fever is a mosquito-borne viral hemorrhagic disease endemic in parts of Africa and Latin America, and although an effective live attenuated vaccine exists, its use is limited by safety and eligibility restrictions. Moreover, large outbreaks continue to expose critical challenges, such as an insufficient vaccine supply, reliance on fractional dosing, and slow and difficult-to-scale manufacturing processes. Here, we report the design, development and in vivo immunogenicity of multiple yellow fever virus (YFV) antigen constructs based on the pre-membrane (prM) and envelope (E) proteins—with or without the transmembrane domain (TM or ΔTM)—delivered using the ChAdOx1 adenoviral vector. **Methods**: Four ChAdOx1 YF vaccines were developed, and immunogenicity was evaluated. The efficacy of the full-length YF envelope vaccine was also tested in Balb/c mice. **Results/Conclusions**: In contrast to previously described orthoflavivirus vaccines on the same platform, the full-length antigen elicited superior immunogenicity and conferred protection against intracranial challenge with the YF17D virus in mice. Notably, this protection was comparable to that induced by the licensed YF17D vaccine, highlighting the promise of this platform as a next-generation yellow fever vaccine candidate.

## 1. Introduction

Yellow fever (YF) is a viral hemorrhagic disease caused by the yellow fever virus (YFV)—an orthoflavivirus transmitted primarily through the bites of infected Aedes or Haemagogus mosquitoes. The virus is endemic in nearly 50 countries, predominantly across sub-Saharan Africa and Latin America. Although a highly effective yellow fever vaccine (YF17D) developed in the 1930s has been instrumental in controlling epidemics, its live-attenuated nature imposes important limitations. These include contraindications for pregnant and lactating women, immunocompromised individuals, and people with hypersensitivity to chicken egg proteins [1].

Yellow fever outbreaks—commonly occurring in Africa—lead to over 100,000 severe cases per year [2], resulting in the challenge of rapidly scaling up the licensed “legacy” and broadly used 17D live-attenuated yellow fever vaccine; this is difficult due to the laborious and regulated manufacturing strategy. During the Angola outbreak in 2016, the lack of vaccine availability prevented control of the spread of the virus [3], prompting regulators and the WHO to establish a fractional dosage to manage vaccine shortages. The recommended one-fifth fractional dose of the standard dose varies in potency from two to five times higher [4,5], depending on the vaccine manufacturer used, which limits the generalizability of the findings. Several clinical trials were performed to establish the minimum fractional dose that induces an immune response equivalent to a full dose [4,6]. This variability in vaccine potency may result in lower seroconversion rates and therefore could affect long-term memory immune responses, YF virus clearance and ultimately protection [7]; however, this has not been established.

Replication-deficient adenovirus-based vector vaccines have been proven to induce a crucial innate immune activation and elicit robust immunogenicity against the encoded transgene expressed. Adenovirus vectors have been tested in several hundred clinical trials, in some cases leading to licensure [8]. During the COVID-19 pandemic, the replication-deficient chimpanzee adenovirus ChAdOx1-nCov-19/AZD1222/Vaxzevria vaccine developed by Oxford/AstraZeneca was initially the most used world-wide, demonstrating an established safety profile, low cost in production and manufacture, easy scalability to an industrial level, and long-term stability at standard refrigerator temperatures [8]. Therefore, the ChAdOx1 adenoviral vector has proven to be a valuable and reliable vaccine platform in emergency situations such as outbreaks.

The transgene-specific induction of adaptive immune responses after adenoviral-vector vaccine immunizations depends on innate immune activation, adenovirus tropism, and levels of transgene expression [9,10,11]. Many studies in mice using non-replicative adenovirus-based vectors have demonstrated that both the magnitude and duration of transgene expression shape the maintenance and phenotype of cellular and/or humoral immune responses [10,12,13]. A replication-competent adenovirus can generate up to 100-fold-higher antigen expression than a replication-deficient adenovirus, resulting in more persistent immunological responses [14,15]. Its safety profile has not yet been demonstrated in clinical settings; therefore, achieving such levels of expression has been a major focus of many vaccinology research groups—including ours—by using the ChAdOx1 platform. Moreover, the use of this platform against the YFV would overcome the manufacturing challenges of the current egg-based vaccine.

For most orthoflaviviruses, the envelope glycoprotein (E), which is the major component of the viral surface glycoprotein shell, is the main antigen target used in vaccine design. Moreover, epitopes within E are the most frequently directed by strong neutralizing and protective antibody responses induced by natural infection or vaccination [16]. Previously, Lopez-Camacho et al. [17] showed that a ChAdOx1 vaccine platform, expressing the Zika virus (ZIKV) pre-membrane (prM) domain together with the E gene sequence without the C-terminal transmembrane domain (TM), positively modulated the immune response against the E protein, by inducing a higher and longer maintained humoral response associated with a stronger efficacy in mice, hypothesizing that this was due to an improvement in antigen secretion.

In this present study, we aim to evaluate whether the inclusion of the pre-membrane protein and inclusion or deletion of the C-terminal transmembrane domain in the envelope protein expressed from the ChAdOx1 vaccine platform might also modulate immunogenicity and efficacy when applied to yellow fever virus. We demonstrate that the full-length antigen comprising prM, E and C-term-TM elicits the strongest and most durable immunogenicity, as well as significantly reducing the YFV viral load, achieving efficacy comparable to the licensed YF17D vaccine. Given the numerous advantages of the clinically proven ChAdOx1 platform, the ChAdOx1 YFprME vaccine may represent a robust candidate for further clinical evaluation.

## 2. Materials and Methods

### 2.1. Chemicals and Plasticware

Unless specified otherwise, all the culture media, reagents and plasticware used in this work were supplied by Thermo Fisher Scientific (Waltham, MA, USA).

### 2.2. Adenoviral Vaccine Vectors

**Figure 1:**
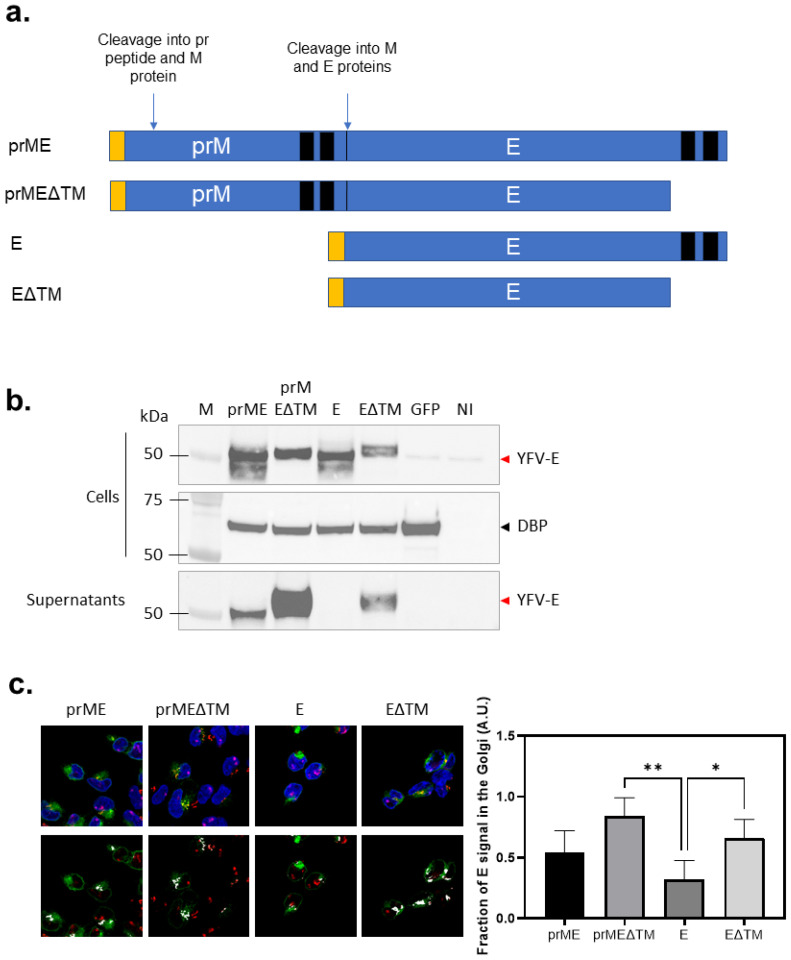
Vaccine design and antigen expression: (**a**) A schematic representation of the yellow fever antigen designs on the vaccines used. The yellow box represents the tPa signal sequence, the black box the transmembrane domain, and the blue box the anchored-membrane envelope glycoprotein. Based on the YF polyprotein strain 17D; prME is amino acids 121 to 778; PrMEΔTM is amino acids 121 to 731; E is amino acids 286 to 778; EΔTM is amino acids 286 to 731). (**b**) YFV envelope antigen expression in HEK-293A cells according to Western blots. The antigen was detected using an anti-YFV-E polyclonal antibody in both cell lysates and immunoprecipitated supernatants. Red arrows corresponds to YFV envelope protein. Black arrow corresponds to DBP, a viral protein, used as a ChAdOx1 infection control. Representative of three independent experiments. From left to right: M, marker; prME, ChAdOx1 prME; prMEΔTM, ChAdOx1 prMEΔTM; E, ChAdOx1 E; EΔTM, ChAdOx1 EΔTM; GFP, ChAdOx1 GFP; NI, not infected. The full blot is included in Figure A1, and the density reading and intensity ratios are in Table A1. (**c**) Confocal immunofluorescence microscopy of infected T-Rex™-293 cells expressing the four YFV-E antigens to assess the cellular distribution (**top**). These are representative confocal images where the fluorescence signal of the envelop E proteins is shown in green, cis Golgi (GM130) in red, and the nucleus (DAPI) is shown in blue. The colocalization of the YFV envelope protein with Golgi (**bottom**) is shown in white. The scale bar represents 5 μm. The graph represents the fraction of viral protein signal associated with the Golgi, calculated as the integrated viral protein fluorescence intensity within the Golgi ROI divided by the total cellular viral protein intensity. Bars indicate mean ± SD (n = 4 images per condition in which 3 to 5 ROIs were analyzed). Data were analyzed with an ordinary one-way ANOVA with Tukey’s multiple-comparison test (ChAdOx1-YFprMEΔTM vs. ChAdOx1-YFE mean difference= 0.597, 95%CI [0.1812, 0.8569], ** *p* = 0.0031; ChAdOx1-YFE vs. ChAdOx1-YFEΔTM mean difference = −0.3385, 95%CI [−0.6757, −0.001387], * *p* = 0.0490).

We followed a similar vaccine design to Lopez Camacho et al. [17]. Four different vaccine constructs were developed containing either pre-membrane and envelope (prME) protein or envelope protein alone (E), with or without the envelope C-terminal 59-amino acid transmembrane domain (TM or ΔTM) (Figure 1). Nucleotide sequences were derived from YFV strain 17D (GenBank accession number JX949181.1). The nucleotide sequences were human codon-optimized, and runs of more than 4 consecutive bases were removed, before they were synthesized by GeneArt (Thermo Fisher Scientific, Life Technologies, Oxford, UK). All constructs included a tPA leader sequence on the 5′ end of the YFV protein sequence. YFV protein ORFs (based on the YF strain 17D polyprotein; prME is amino acids 121 to 778; PrMEΔTM is amino acids 121 to 731; E is amino acids 286 to 778; EΔTM is amino acids 286 to 731) were subcloned into gateway recombination shuttle plasmids, using KpnI and Not-I restriction enzymes to insert the ORF between a modified human cytomegalovirus immediate early promoter, with intron A and a tetracycline-regulated operon, and a bovine growth hormone poly A signal sequence. The resulting shuttle plasmids were used to insert the antigen expression cassettes into the E1 loci of the ChAdOx1 Gateway Destination plasmid (bacterial artificial chromosome (BAC) containing ChAdOx1 genome) by Gateway recombination as previously described [18]. Linearized viral vector genomes excised from the resulting BACs were transfected into T-REx-293 cells (Invitrogen, Birmingham, UK) using Lipofectamine 2000 (Invitrogen, UK), and the virus vectors were rescued and propagated. The virus vectors were purified by CsCl gradient ultracentrifugation, and the virus was titrated as previously described [18].

### 2.3. Immunofluorescence Microscopy

T-Rex™-293 cells (Thermo Fisher Scientific, UK) grown on glass coverslips of 13 mm diameter were infected at a MOI of 10 IU/cell with ChAdOx1 viruses and incubated at 37 °C under 5% CO_2_ for 18 h. After 18 h, the cells were fixed with Image-IT™ Paraformaldehyde 4% Fixative solution (Thermo Scientific, UK) for 10 min at room temperature (RT), followed by permeabilization with a PBS buffer containing 0.1% *v*/*v* Triton-X100 in PBS. The YFV E proteins were visualized using the Yellow Fever Virus antibody 2D12 (0G5) (Bio-Rad, Watford, UK), and the Golgi apparatus was visualized using the Anti-GM130 antibody [EP892Y] cis-Golgi Marker (Abcam, Cambridge, UK), both at dilutions of 1:100 in a PBS buffer containing 2% *v*/*v* FBS (blocking buffer), for 1 h at RT. Following several washes with blocking buffer, the cells were subsequently incubated at RT for 1 h with a goat anti-mouse IgG (H + L) cross-adsorbed secondary antibody, Alexa Fluor™ 488, and a goat anti-rabbit IgG (H + L) highly cross-adsorbed secondary antibody, Alexa Fluor™ Plus 647 (Invitrogen, UK), both diluted 1:250 in blocking buffer and with DAPI nucleic acid stain at a dilution of 1:1000 also in blocking buffer. After several washes in blocking buffer, the coverslips were mounted on a glass slide with Anti-Fade Fluorescence Mounting media (Abcam, UK). Confocal laser scanning of fluorescence was performed using an FV4000 Laser Confocal Microscope (Evident Scientific, Hamburg, Germany). The images were analyzed using ImageJ software (ImageJ/Fiji 1.46) [19]. The Golgi association of YFV E proteins was also quantified using ImageJ/Fiji 1.46 (ImageJ). The confocal images were split into individual channels corresponding to the viral protein signal and the Golgi marker GM130. The background was subtracted from both channels using a rolling-ball algorithm with a radius of 30 pixels. For each cell, a whole-cell region of interest (ROI) was manually defined on the viral protein channel. Golgi regions were segmented from the GM130 channel using automatic intensity thresholding (Moments method), followed by conversion to a binary mask. The fraction of viral protein signal associated with the Golgi (FG) was calculated as the integrated fluorescence intensity of the viral protein within the GM130-defined Golgi ROI divided by the total integrated fluorescence intensity of the viral protein within the whole-cell ROI. This analysis was performed on four independent images per condition, in which 3 to 5 ROIs were analyzed. Quantitative values are reported as mean ± SD.

### 2.4. Immunoprecipitation and Western Blotting

HEK-293A cells were infected at a MOI of 5 IU/cell with ChAdOx1 viruses and incubated at 37 °C under 5% CO_2_ for 24 h. After 24 h, the cells and supernatants were harvested. The cells were pelleted down by centrifugation at 1000× *g* for 10 min at RT. The cells were lysed with 1 volume of 1%w/v SDS for 10 min at RT, followed by 9 volumes of RIPA buffer (Sigma; UK). The cell lysates were clarified by centrifugation at 17,000× *g* for 20 min at 4 °C. E proteins were immunoprecipitated from the supernatants using Dynabeads™ Protein G (Invitrogen) coupled to the Yellow Fever Virus Antibody c2D12 (0G5) (Bio-Rad) following the manufacturer’s protocol. 4X LDS Sample Buffer (Invitrogen) and 100 mM DTT were added to the cell lysates and to the immunoprecipitated supernatants before being boiled at 100 °C for 10 min. Equal amounts of cell lysates and immunoprecipitated supernatants were resolved by SDS-PAGE and transferred onto nitrocellulose membranes. Blots were blocked with Pierce Protein Free T20 (Thermo Fisher Scientific, UK) Blocking Buffer and incubated with either the Yellow Fever Virus Envelope Protein (strain 17D vaccine) Recombinant Rabbit Monoclonal Antibody (Invitrogen, UK) or the Rabbit anti-Human adenovirus C serotype 5 (HAdV-5) (Human adenovirus 5) DBP Polyclonal antibody (Cusabio, Houston, TX, USA), followed by incubation with an anti-rabbit HRP-conjugated antibody (Agiletn technologies, Cheshire, UK). The chemiluminescence (Clarity™ Western ECL Substrate, BioRad) was visualized using a BioRad Chemidoc MP Imaging System.

### 2.5. Virus for Efficacy Testing

A YF17D strain, Stamaril^®^ (Sanofi-Pasteur, Lyon, France), was passaged three times in Vero E6 cells before use. Virus titers were determined by plaque assays on BHK-21J cells, expressed as plaque-forming units, PFU/mL.

### 2.6. Animals and Immunizations

All animal experiments at the University of Oxford were conducted in compliance with the UK Animals (Scientific Procedures) Act 1986 under project license number PP2352929, granted by the UK Home Office. Approval was also obtained from the Animal Welfare and Ethical Review Body (AWERB) at the University of Oxford.

Animal experiments at the Rega Institute for Medical Research, KU Leuven (University of Leuven, Belgium), were conducted strictly according to the required Belgian guidelines for animal experimentation and guidelines of the Federation of European Laboratory Animal Science Associations (FELASA), under the project license number P100/2019 granted by the Animal Research Center and Ethics Committee of KU Leuven.

These studies adhered to the Animal research Reporting of In vivo experiments (ARRIVE) guidelines following the principles of the 3Rs (replacement, reduction and refinement).

At the University of Oxford, the mice were housed in individually ventilated cages under specific pathogen-free (SPF) conditions, and maintained at a constant temperature and humidity, with a 13:11 light–dark cycle (lights on from 7 a.m. to 8 p.m.). Inbred female Balb/cOlaHsd (Balb/c) mice were obtained from the commercial suppliers Envigo RMS UK Ltd. (Loughborough, UK) and Inotiv UK (Blackthorn, UK). Upon arrival, the animals were randomly allocated into experimental groups. After one to two weeks of acclimation, for single-dose and homologous prime-boost regimens, 6–8-week-old mice were immunized intramuscularly (IM) in the tibialis muscle with 1 × 10^8^ infectious units (IU) of one of the ChAdOx1 YF vaccines, delivered in a total volume of 50 μL. Vaccine administration was carried out under general isoflurane (IsoFlo^®^) anesthesia, ensuring full unconsciousness, verified by the absence of a pedal withdrawal reflex. Serum and spleens were collected for the analysis of humoral and cell-mediated immunity, respectively. All mice were humanely sacrificed at the conclusion of each experiment (four weeks after the final immunization) using an approved Schedule 1 method, i.e., exsanguination via cardiac puncture under general anesthesia followed by cervical dislocation.

At KU Leuven, 5–6-week-old female BALB/c mice of approximately 20 g were ear-tagged and randomized into different groups. Mice received either the ChAdOx1-YFprME vaccine (IM) as a test item or the YF17D vaccine (intraperitoneal, IP) as a positive control. One group received a homologous ChAdOx1 prime-boost immunization four weeks apart (1 × 10^8^ IU). A non-vaccinated group, receiving the same volume of culture medium (IP; mock/control), was used to test the efficiency of the challenge. Mice were bled from the facial vein before challenge to test neutralizing antibodies by the SNT (serum neutralization test). Twenty-eight days after the second immunization, mice received an intracranial (i.c.) injection with 30 μL containing 3 × 10^3^ PFU of YF17D while deeply anesthetized by using an anesthetic combination of 0.4 μL of atropine, 0.4 μL of ketamine and 0.8 μL of xylazine per gram of body weight. All mice were monitored daily for signs of disease and weight change. Sick mice were euthanized at humane endpoints, based on morbidity (hind limb paralysis, weakness and ruffled fur) or weight loss of more than 15% (warranting refinement of the experiment and implementation of the 3R principle). Five to seven days after virus inoculation (when the mock-vaccinated groups generally reached humane endpoints), the peak of infection could be detected, and all mice were sacrificed.

### 2.7. Enzyme-Linked Immuno-Spot Assay (ELISpot)

Cellular immune responses were assessed using an ELISpot assay. The 15-amino-acid-long peptides, overlapping by 11 residues, were dissolved in dimethyl sulfoxide (DMSO) to a concentration of 100 mg/mL and grouped into four pools covering the YF preM and E proteins. The 131 all-L-amino acid peptides spanning the YF preME antigen sequence used for the ELISpot assays reported here were synthesized by Pepscan (Lelystad, The Netherlands).

Single-cell suspensions of spleens were prepared by processing tissues with C tubes and a gentleMACS dissociator (Milteny Biotec, Bergisch Gladbach, Germany), followed by filtration through 70 μm cell strainers. Red blood cells were lysed using Ammonium–Chloride–Potassium (ACK) solution, and splenocytes were subsequently resuspended in RPMI complete medium, i.e., supplemented with 10% fetal bovine serum (FBS) and 1% pen/strep antibiotics. Cell viability was assessed using a Countess™ 3 Automated Cell Counter, and samples with viability below 80% were excluded from the analysis. The cells were stimulated with peptide pools spanning the PreM or E antigens’ regions at a final concentration of 2 μg/mL in Immobilon^®^-P PVDF (IPVH) membrane plates (Millipore, Burlington, MA, USA) pre-coated with 5 μg/mL anti-mouse IFN-γ antibody AN18 (Mabtech, Nacka Strand, Sweden). The plates were incubated at 37 °C with 5% CO_2_ in a humidified incubator for 18–20 h. Interferon gamma (IFN-γ) spot-forming cells were detected by staining the membranes with biotinylated anti-mouse IFN-γ antibody (1 mg/mL), followed by streptavidin–alkaline phosphatase (1 mg/mL). Spot development was performed using an alkaline phosphatase (AP) conjugate substrate kit (Bio-Rad, Watford, UK). The number of spots was quantified using an AID ELISpot reader (Autoimmun Diagnostika GmbH, Straßberg, Germany) and AID software 7.0 (Autoimmun Diagnostika GmbH).

### 2.8. Enzyme-Linked Immunosorbent Assay (ELISA)

Humoral immune responses were evaluated using an ELISA. MaxiSorp ELISA plates were coated with 50 μL of 2 μg/mL YFV E protein (The Native Antigen, Kidlington, UK) diluted in PBS and incubated overnight at 4 °C. Following incubation, the plates were washed six times with PBS containing 0.05% Tween-20 (PBS-Tween) and blocked with 100 μL per well of Casein blocking buffer for 1 h at room temperature. Mouse sera were subsequently diluted in Casein blocking buffer, with the starting dilution determined by the immunization regimen and sampling time point, and serially diluted threefold down the plate. The plates were incubated for 2 h at room temperature, followed by washing and a 1 h incubation with goat anti-mouse whole IgG conjugated to alkaline phosphatase at room temperature. The plates were then developed by adding p-nitrophenyl phosphate (Sigma, London, UK) at 1 mg/mL in diethanolamine substrate buffer. The optical density (OD) values were measured at 405 nm using a Bio-Tek ELx800 Microplate Reader (BioTek Instruments, Winooski, VT, USA). For each sample, ELISA units were calculated using the OD values and the parameters of the standard curve, with the results expressed in arbitrary units (AU). Statistical analyses were performed to determine significant differences in immune responses across groups.

### 2.9. Serum Neutralization Test for Neutralizing Antibody Titer

The quantitative detection of YFV-specific neutralizing antibodies (nAb) has been described and validated in great detail before [20]. YFV nAbs were determined using mCherry-tagged YF17D reporter viruses. All reagents were prepared and methods were performed accordingly [21,22,23].

### 2.10. Viral RNA Titration in Different Organs and Gene Cytokine Expression in the Liver

Total RNA was extracted from homogenized mouse tissues using the NucleoSpin RNA virus 250 kit (Macherey Nagel, Belgium) according to the manufacturer’s protocol. Viral RNA copies were determined by RT-qPCR using a forward primer (5′-CAC GGC ATG GTT CCT TCC A-3′), a reverse primer (5′-ACT CTT TCC AGC CTT ACG CAA A-3′) and a probe (5′-FAM-CAG AGC TGC AAA TGT C-3′) derived from the YFV non-structural gene 3 (NS3). RT-qPCR was performed using the ABI 7500 Fast Real-Time PCR System (Applied Biosystems, Branchburg, NJ, USA). For absolute quantification, standard curves were generated using five-fold dilutions of a cDNA plasmid template (plasmid pShuttle/YF17D) of known concentration [21,22].

To profile the gene expression for pro-inflammatory cytokines (IP-10, OAS-1) in the liver [21,22], RT-qPCR was performed on a Light Cycler 96 platform (Roche, Machelen, Belgium) using the iTaq Universal Probes One-Step RT-qPCR Kit (BioRad) with primers and probes. The relative RNA fold-change was calculated by the 2^−ΔΔCq^ method using the housekeeping gene GAPDH for normalization.

### 2.11. Statistical Analyses

All statistical analyses were performed using GraphPad Prism version 9.2.0 (GraphPad Software, San Diego, CA, USA). Data were first tested for normality before being analyzed using either a two-way ANOVA followed by Šídák’s multiple-comparison test, or a one-way ANOVA with Tukey’s multiple-comparison test (specified in each figure legend). Significant differences are indicated directly on the graphs, denoted as * *p* < 0.05, ** *p* < 0.01, *** *p* < 0.001, and **** *p* < 0.0001. The mean differences, 95% confidence intervals, and specific *p* values of statistically significant comparisons are indicated in the figure legends.

## 3. Results

### 3.1. Design and Expression of Yellow Fever Antigen

We developed ChAdOx1-vectored vaccines following the carboxyl-terminal transmembrane (TM) depletion strategy, described by Lopez-Camacho et al. [17], with or without the inclusion of the pre-membrane (prM) region (Figure 1a). Four different constructs were evaluated for protein expression: prME, prMEΔTM, E and EΔTM. Envelope protein expression was readily detected in cell lysates by Western blotting (Figure 1b); however, the immune precipitation of cell culture supernatants was required to enrich secreted surface proteins for detection (Figure 1c). Clear differences were observed among the constructs: the depletion of the C-terminal TM positively impacted the secretion of the proteins. In contrast, the surface antigen lacking the prM domain was not detected in the supernatants; however, the removal of the C-terminal TM resulted in the appearance of detectable protein (Figure 1b), suggesting an alteration in the E specific cellular secretory pathway when expressed from a ChAdOx1 E vector in mammalian cells. The apparent molecular weights of the E constructs did not fully correspond to their predicted sizes based on amino acid sequences. This might be explained by differences in N-linked glycosylation, since proteins receive high-mannose glycans in the ER that can be further modified into complex glycans during trafficking through the Golgi apparatus [24,25]. We aimed to evaluate whether the ΔTM constructs were more efficiently secreted, and therefore more extensive Golgi processing, which could alter their electrophoretic mobility on SDS-PAGE. Consistent with this, we performed a treatment with PNGase F (peptide N-glycosidase F), prompting normalization of the migration of the different constructs to the same size, indicating that differential glycosylation accounts for the observed band shifts.

We next evaluated the cellular distribution of the surface proteins by confocal fluorescence microscopy (Figure 1c). E protein staining exhibited a more diffuse pattern when the C-terminal TM was deleted, compared to the full-length antigen, irrespective of the presence of the prM domain. Colocalization analysis of the envelope protein with the Golgi marker (GM130) displayed a clear reduction with the E construct, corroborating the Western blot results and indicating a disruption of the envelope protein secretion pathway [26].

### 3.2. Robust and Long-Lasting Serum Antibody Responses

**Figure 2:**
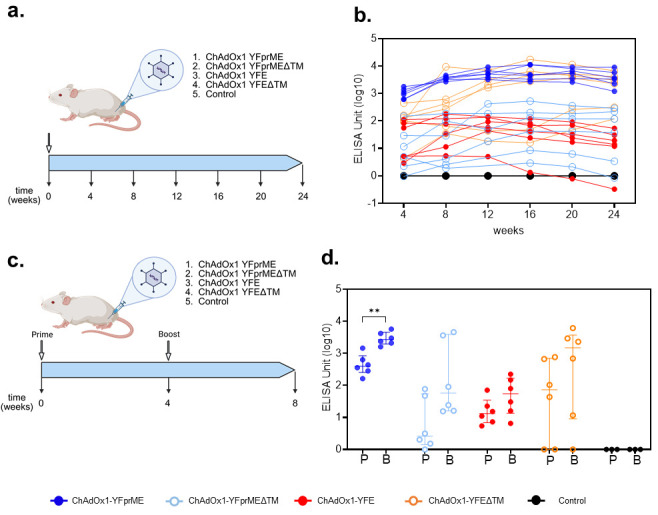
Experimental design and antibody titers after immunization. (**a**) Four groups of six mice (a group per vaccine tested) and a control group (PBS, n = 3) were intramuscularly vaccinated and bled every four weeks for 6 months (figure constructed on Biorender.com). (**b**) IgG anti-YF envelope protein (E) titers from sera of mice immunized with ChAdOx1 vaccines, withdrawn every four weeks for a six-month period. Each symbol (circle) represents one animal. Data represent one single experiment. Graph was plotted with log axis to improve visualization. (**c**) Four groups of six mice (a group per vaccine tested) and a control group (PBS, n = 3) were intramuscularly vaccinated with homologous vaccines four weeks apart and bled four weeks after immunization (figure constructed on Biorender.com). (**d**) IgG anti-YF envelope protein (E) titers from sera of mice immunized with ChAdOx1 vaccines, withdrawn four weeks after prime (P) or prime-boost (B) immunization. Each symbol (circle) represents one animal. Scatter dot plot representations with lines showing medians and interquartile ranges are presented. Data represent two independent experiments. Graph was plotted with log axis to improve visualization. Data were analyzed with two-way ANOVA with Šídák’s multiple-comparison test (ChAdOx1-YFprME mean difference = −2618, 95%CI [−4498, −738.3], ** *p* = 0.0027).

The ChAdOx1 Zika vaccine previously developed [17], comprising the envelope protein with the inclusion of the prM region and the deletion of the C-terminal TM region, induced the highest anti-Zika virus envelope protein antibody titers, whose durability was maintained up to 9 months. Since serum antibody IgG levels are considered a serological correlate of protection for YF [7,21], we aimed to evaluate and follow the different ChAdOx1 YF vaccine designs’ humoral responses for up to 6 months. Balb/c mice were immunized with a single dose of 10^8^ IU with one of each of the four different vaccine designs (Figure 2a).

The deletion of the C-term TM region (C-terminal 59 amino acids of E protein) drastically impacted the induction of anti-envelope IgG titers compared to the full-length (prME) protein vaccine (Figure 2b). Likewise, the ChAdOx1-YFprME vaccine induced the highest levels of anti-YF envelope protein IgG, with a median value titer of over 103 ELISA units over the six-month period, compared to ChAdOx1-YFprMEΔTM or even ChAdOx1-YFE, both with median titers of around 10^1^ over the same six-month period. Interestingly, when the envelope antigen lacked the prM domain together with the C-term TM region, antibody titers increased to approximately the same titer level of the ChAdOx1-YFprME; however, the response was more variable, with the response elicited not being consistent in all the animals. No or very low detectable levels of neutralizing antibodies were found after vaccination with the vaccines tested at any time point tested.

Due to the lack of neutralizing activity detected after prime only, we then aimed to boost the humoral response with an extra homologous immunization four weeks after prime immunization (Figure 2c). An enhancement of the antibody titers was detected for all the vaccines tested, whereby the ChAdOx1-YFprME vaccine was the only one where booster immunization induced a significant difference (one log increase), mostly due to the dispersed response detected in the other vaccine groups (Figure 2d). Neutralizing antibodies remained below the limit of detection.

### 3.3. YF Pre-Membrane and Envelope Protein Specific Cellular Responses

**Figure 3:**
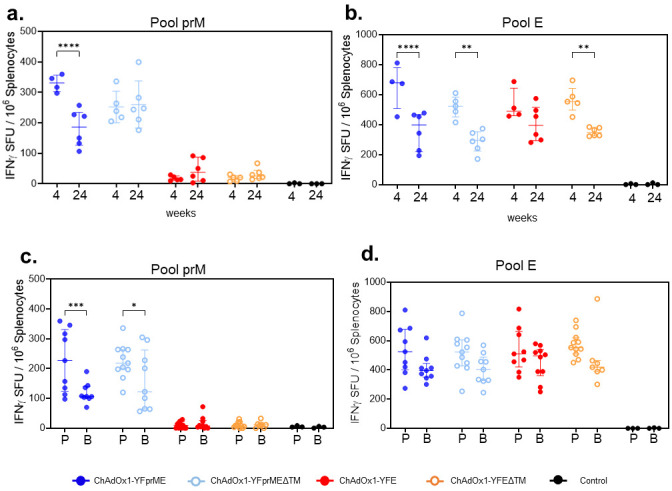
IFNγ T cell responses after prime or prime-boost vaccination. IFNγ-secreting spot-forming units (SFU) were enumerated in the spleen after 4 and 24 weeks after single immunization (**a**,**b**), or 4 weeks after prime or prime-boost immunizations (**c**,**d**). Four groups of six mice (a group per vaccine tested) and a control group (PBS, n = 3) were evaluated. Splenocytes were stimulated with a pool of peptides covering prM (**a**,**c**) and E protein (**b**,**d**). (**a**,**b**) correspond to single experiments, while (**c**,**d**) correspond to a compilation of data from two independent experiments. Cell viability was assessed, and if viability was less than 80%, results were not included. Each symbol (circle and square) represents one animal. Scatter dot plot representations with lines showing medians and interquartile ranges are presented. (**a**) Data were analyzed with two-way ANOVA with Šídák’s multiple-comparison test (ChAdOx1-YFprME mean difference = 147.3, 95%CI [72.96, 221.6], **** *p* < 0.0001). (**b**) Data were analyzed with two-way ANOVA with Šídák’s multiple-comparison test (ChAdOx1-YFprME mean difference = 295.8, 95%CI [132.5, 459.0], **** *p* < 0.0001; ChAdOx1-YFprMEΔTM mean difference = 230.8, 95%CI [77.61, 384.0], ** *p* = 0.0011; ChAdOx1-YFEΔTM mean difference = 214.8, 95%CI [61.61, 368.0], ** *p* = 0.0026). (**c**) Data were analyzed with two-way ANOVA with Šídák’s multiple-comparison test (ChAdOx1-YFprME mean difference = 109.5, 95%CI [41.66, 177.4], *** *p* = 0.003; ChAdOx1-YFprMEΔTM mean difference = 65.17, 95%CI [0.4579, 129.9], * *p* = 0.047). E, envelope protein; PrM, pre-membrane; SFU, spot-forming units; P, prime; B, boost.

ChAdOx1 is well known to elicit a robust T cell response in humans [27] and different animal models [28]; however, the licensed YF17D vaccine is known to induce rather weak T cell responses in mouse models unless an IFNAR-blocking antibody is used [21]. We evaluated cellular responses by IFNγ ELISpots after prime vaccination in the spleen against a pool of peptides comprising either the length of prM or the E domain. Four weeks after prime vaccination, all the vaccines induced a response over 500 IFNγ spot-forming units (SFU) per million cells against the envelope protein pool of peptides, with ChAdOx1-YFprME inducing the strongest cellular response, although not statistically significant compared to the other vaccines (Figure 3b). PrM induces a modest response, with ChAdOx1-YFprME being the one with a more prominent response but not statistically different to ChAdOx1-YFprMEΔTM (Figure 3a). Twenty-four weeks after prime vaccination, a decrease in cellular responses was detected in all the vaccine groups, as has been frequently detected in humans [29] and mouse models [30] due to an effector T cell contraction phase following antigen and inflammation fading and the generation of a memory response [28].

A prime-boost (P-B) strategy is the usual rational approach to enhancing immunogenicity, so we then evaluated cellular responses after boost vaccination. Four weeks after boost immunization, there were slight reductions in the cellular responses in all the groups with the different vaccines tested, but these were not statistically significant when stimulated with the E pool of peptides (Figure 3d) as compared to the prM domain pool (Figure 3c).

The presence or absence of either the C-term TM region or the prM domain was not found to have any effect in cellular immune responses, although the full antigen constructs trended towards slightly higher responses.

### 3.4. Protective Efficacy of ChAdOx1-YFprME Immunization After Lethal Intracranial YFV Challenge

**Figure 4:**
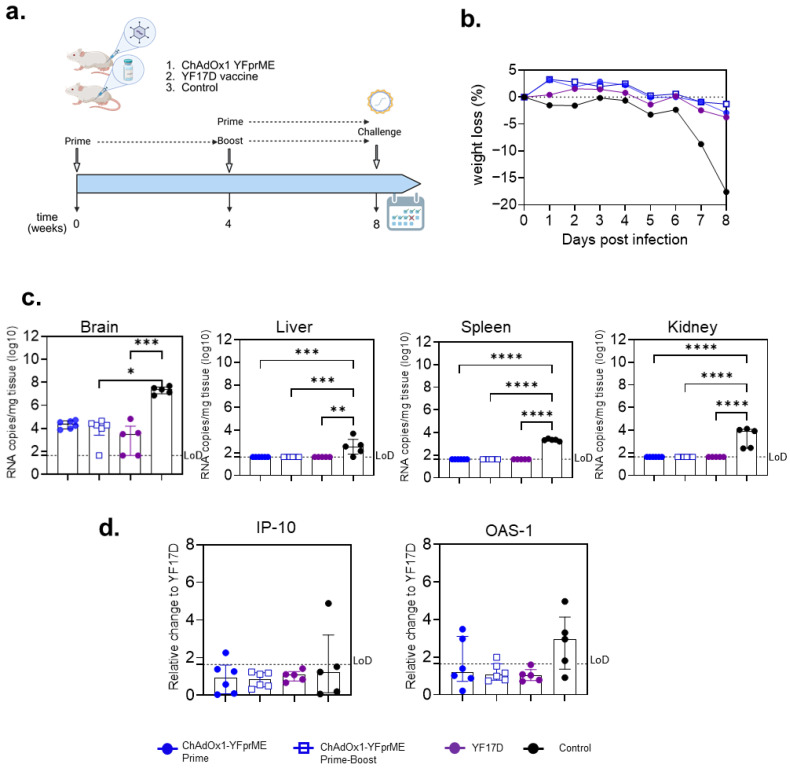
Either prime or homologous prime-boost ChAdOx1 YFprME vaccination induces protection against yellow fever. (**a**) Three groups of five mice (per vaccine/regimen tested) and a control group (media) were either intramuscularly (ChAdOx1 YFprME) or intraperitoneally vaccinated (YF17D, control). Four weeks after the last immunization, an intracranial administration of YF17D virus was performed (figure prepared on Biorender.com). (**b**) Percentage of weight loss of the different groups relative to the weight on the day of challenge. (**c**) Viral load 8 days after challenge expressed as copies of RNA per mg of tissue (log scale). From left to right: brain, liver, spleen and kidney viral load data were analyzed with one-way ANOVA with Tukey’s multiple-comparison test (brain: Control vs. YF17D geometric mean ratio difference = 2.631, 95%CI [1.493, 4.636], *** *p* < 0.0007; Control vs. ChAdOx1-YFprME prime-boost geometric mean ratio difference = 1.694, 95%CI [1.154, 3.414], * *p* = 0.0106) (liver: Control vs. YF17D geometric mean ratio difference = 1.5, 95%CI [1.166, 1.930], ** *p* = 0.0013; Control vs. ChAdOx1-YFprME Prime geometric mean ratio difference = 1.5, 95%CI [1.179, 1.909], *** *p* = 0.0008; Control vs. ChAdOx1-YFprME prime-boost geometric mean ratio difference = 1.5, 95%CI [1.179, 1.909] *** *p* = 0.0008) (spleen: Control vs. YF17D geometric mean ratio difference = 2.039, 95%CI [1.990, 2.090], **** *p* < 0.0001; Control vs. ChAdOx1-YFprME Prime geometric mean ratio difference = 2.039, 95%CI [1.992, 2.087], **** *p* < 0.0001; Control vs. ChAdOx1-YFprME prime-boost geometric mean ratio difference = 2.039, 95%CI [1.992, 2.087] **** *p* < 0.0001) (kidney: Control vs. YF17D geometric mean ratio difference = 2.002, 95%CI [1.580, 2.535], **** *p* < 0.0001; Control vs. ChAdOx1-YFprME Prime geometric mean ratio difference = 2.002, 95%CI [1.596, 2.510], **** *p* < 0.0001; Control vs. ChAdOx1-YFprME prime-boost geometric mean ratio difference = 2.002, 95%CI [1.596, 2.510] **** *p* < 0.0001). (**d**) Relative expression of IP-10 and OAS-1 genes in liver compared to YF17D-vaccinated group. LoD, limit of detection.

Since a noticeable difference among vaccines was found in humoral immune responses, we decided to move forward and evaluate the protective efficacy of the ChAdOx1-YFprME vaccine after either prime or prime-boost immunization. A positive control group with a single immunization with the licensed YF17D vaccine and a negative control group (non-vaccinated, PBS) were included (Figure 4a).

Eight days post infection, mice from the control group developed acute weight loss and progressed to a severe neurological disease with ruffled fur, hunched posture and hind limb paralysis, reaching the humane endpoint. By contrast, none of the mice from the vaccine groups developed neurological signs of disease (Figure 4b) requiring euthanasia, corresponding to 100% survival. Different tissues were then harvested to evaluate viral RNA loads, and a reduction in viral presence was detected in all the vaccinated groups. ChAdOx1-YFprME was equally effective as the YF17D vaccine in clearing the liver, spleen and kidney from YF virus as compared to the control group (Figure 4c), and a significant reduction in virus load in the brain was observed. Remarkably, no differences were found when prime or prime-boost immunization with ChAdOx1-YFprME was compared.

IFN-inducible OAS-1 (2′-5′-oligoadenylate synthase 1) and IP-10 (interferon gamma inducible protein 10) expression, as determined by RT-qPCR, showed an unresolved virus-related inflammatory response in two mice (20%) after a single immunization with ChAdOx1-YFprME, which was found to be different from the control group (80% in the case of OAS-1 expression) (Figure 4d).

NAbs were not found in any of the mice at the time before challenge with YFV in either the ChAdOx1-YFprME- or YF17D-vaccinated groups.

The candidate vaccine ChAdOx1-YFprME in a single immunization induced complete protection against YFV challenge in a small animal model similar to the licensed YF17D vaccine.

## 4. Discussion

Here, we use the broadly distributed and clinically proven ChAdOx1 platform to reveal, for the first time, the importance of keeping the full length of an orthoflavivirus (i.e., YFV) envelope protein, with the prM protein and the TM, for vaccine design, in contrast to the Zika vaccine described by Lopez-Camacho et al. [17].

Immature orthoflavivirus particles contain the transmembrane glycoproteins prM and E enclosing the viral genome as a ribonucleoprotein complex together with the capsid protein. prM functions as a chaperone for folding E into a functional membrane protein, which assembles at the membrane of the endoplasmic reticulum (ER) and is then transported to the cell surface through the trans-Golgi network [31]. Although the molecular interactions among the proteins are crucial for the flavivirus maturation process, each orthoflavivirus species appears to modulate them differently [31]. Moreover, it is also known that viral surface proteins need to accumulate in the appropriate compartment before budding takes place [32]. Previously reported research found that the transmembrane domains of prM or E of YFV are crucial for subcellular localization [33] as well as subviral particle release and infectivity [34]. Our results demonstrate that E protein expression in cell lysates is similar among all the vaccine constructs. In contrast, E protein’s secretion, subcellular expression patterns, and intracellular localization are significantly altered when prM, the C-terminal transmembrane (TM) domain, or both are absent. Generally, prM and E play essential roles in the biogenesis of the orthoflaviviral envelope [35,36]. Co-expression of prM and E is sufficient to drive the formation of non-infectious subviral particles (SVPs) lacking a nucleocapsid core, as demonstrated for YFV [34], tick-borne encephalitis virus [37], and dengue virus [38]. However, subtle mutations or deletions in prM can impair or abolish SVP production. In this context, deletions affecting prM and/or the C-terminal TM region of E may disrupt the proper trafficking and secretion of SVPs. Because SVPs present E protein in a highly ordered, particulate form that efficiently engages B-cell receptors, impaired SVP secretion may reduce B-cell activation and the induction of neutralizing antibodies. Conversely, intracellular retention of E antigen may favor antigen processing and presentation via MHC pathways, thereby promoting T-cell responses directed toward intracellularly expressed antigen. Further studies may need to be performed to address the exact mechanism of release of the YF envelope protein to further explain the variable immune response elicited with the different vaccine constructs. Nevertheless, our data shows that antigen design differences clearly modify immunogenicity, highlighting the need for virus-specific vaccine designs.

NAbs are considered the primary correlate of protection following YF17D vaccination, commonly quantified by neutralization assays with a titer of 1 in 10 or higher [39]. However, while nAbs serve as an established correlate of protection, the precise immune mechanism responsible for protection against yellow fever virus remains not completely defined. The work of Theiler and Smith (1937) [40], who described immunity “as measured by the antibody titer developed”, established nAbs as a correlate of protection rather than proof of antibody-mediated protection per se. Other antibody effector functions, as well as cellular responses (CD4+ and CD8+ T cells), may contribute to YF protective immunity [41,42]. The limited availability of an immunologically relevant, immunocompetent small mouse model for YF disease, as well as the limited resources accessible for other animal models (hamsters and non-human primates), makes it difficult to address other relevant surrogates or mechanisms of protection. Studies in small animal models have shown that the total levels of YF virus-specific IgG antibodies, not only nAb, may suffice to induce protection against a lethal YF17D intracranial challenge [21,43,44]. Our vaccine, ChAdOx1 YFprME, tested in Balb/c mice, induced very high E-specific IgG titers, which persisted over time. However, non-detectable to very low levels of nAb were found following vaccination, similar to those detected with the licensed YF17D vaccine in the same animal model. Despite this, ChAdOx1 YFprME was still able to protect against virus dissemination to different organs to levels comparable to those achieved with the licensed YF17D vaccine, even after a single dose. These findings suggest that protective immunity in this model may not rely exclusively on detectable nAbs. These findings are supported by recent human data [41] and have been further discussed previously [42].

Interestingly, the ZIKV protease NS2B3’s inclusion into a capsid-VLP-based YF vaccine has previously been shown to induce higher VLP secretion and increased levels of nAb [45] in Balb/c mice. The same may hold true for our vaccine constructs, and the lack of nAb might be related to the limited secretion of PrME-derived particles. This observation may suggest the potential need to include a protease in the vaccine construct to possibly facilitate the release of prME from the cellular space and thereby enhance the induction of a higher level of nAb.

In addition to humoral responses, we have also demonstrated that the ChAdOx1 YFprME vaccine also showed a strong IFNγ-producing cellular response against prM and E protein which may be comparable to that elicited by the licensed YF vaccine in humans [46,47], although clinical trials would be necessary to confirm it. Together, these observations support the idea that vaccine-induced cellular immunity may contribute to protection even though neutralizing antibodies are low or non-detectable.

Although the intracranial YF17D challenge model used here is severe and does not fully replicate the natural, mosquito-borne route of infection, it provides a robust measure of vaccine-mediated protection while avoiding the BSL3 handling of wild-type virus [48]. The high challenge dose employed (3 × 10^3^ PFU) likely represents a conventional way for severe disease to occur rather than the natural course of infection, highlighting that protection in this model may primarily reflect the vaccine’s ability to prevent severe pathology.

A limitation of our study is that only the full-length ChAdOx1 YFprME construct was taken forward into the intracranial challenge experiments. Considering the 3R guidelines (replacement, reduction and refinement) of animal use, mostly supported by the principle of reduction, the other vaccine designs were not tested in the challenge due to a combination of the inferiority in humoral immunogenicity observed in earlier experiments, and also resource constraints. While this approach allowed us to focus on the most promising construct, it should be noted that the results cannot be fully generalized to all antigen designs. Nevertheless, the comparative immunogenicity data presented for the other constructs provide useful insight into how modifications of prM and E influence antigen trafficking, subviral particle secretion, and immune responses.

Although there is already a licensed YFV vaccine available, shortages due to limitations of vaccine production during outbreaks is of great concern. Fractional dosing (1/5-dose) of the yellow fever vaccine is restrictively recommended in emergency outbreak situations; however, several limitations have arisen during several clinical trials evaluating the fractional dose [49,50]: nAb titers similar to the full dose are the only endpoint for evaluating effectiveness, and no prevention of infection has been evaluated; nAbs are measured in PRNTs but frequently not calibrated using a reference standard; every WHO prequalified vaccine (from different manufacturers) has different potency, and therefore, the final fractional dose and consequent results differ among studies; a minimum dose of 500 IU is established for adults in some studies, which is below the current WHO minimum potency specification, and is not translatable to all ages [4,51]. Moreover, from an immunology perspective, a recent study comparing different immunological parameters after a fractional and standard dose vaccination showed a distinct induction of immune response profiles without significantly impacting the titers of nAb, assuming possible differences in the kinetics of the immune response [52]. All these limitations and differences might be detrimental to a long-lasting immunity or to protection against YF infection, but this has not been studied.

ChAdOx1’s safety, tolerability, immunogenicity and efficacy have been successfully evaluated world-wide during the COVID-19 pandemic. Moreover, the first commercial batch of this vaccine platform could be achieved in less than 100 days by producing 1000 doses per liter of bioreactor capacity per day, with a cost of less than $1 per dose of drug substance [46]. We therefore propose our ChAdOx1 YFprME vaccine as a novel candidate YF vaccine to fight YFV outbreaks and to further manage YF17D vaccine shortages. This vaccine could serve as a stockpile backup, provide an alternative for populations in whom live-attenuated YF17D vaccination is contraindicated, and facilitate rapid surge capacity during outbreaks. A key next step includes the evaluation of protective efficacy in Syrian hamsters or non-human primates, naturally susceptible to YFV infection and disease, which will also guide the design of clinical trials to establish safety, immunogenicity and protective efficacy in humans.

## Figures and Tables

**Figure A1:**
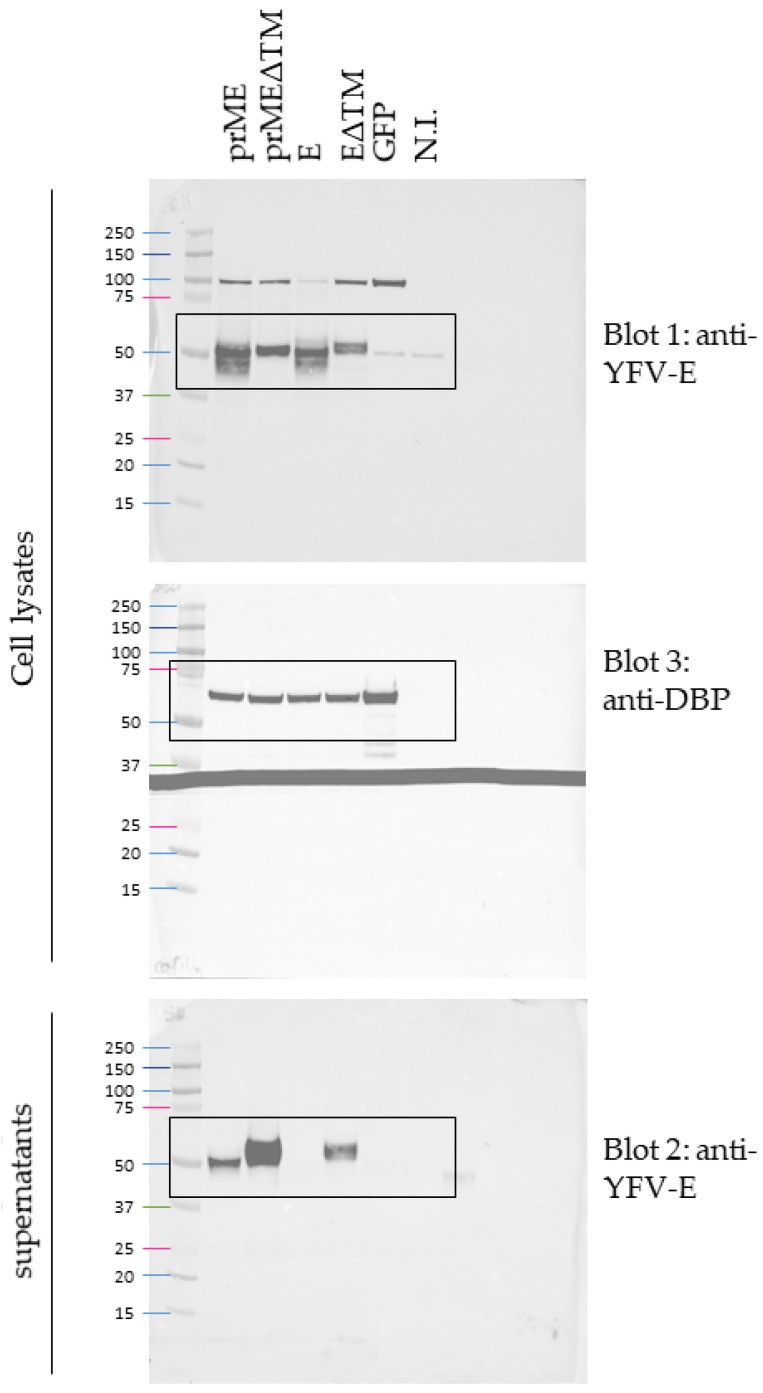
Uncropped images from the main Figure 1. Rectangles represent the cropped image from the main Figure 1. The Blot 3 membrane was cut in half to be able to blot it with two different antibodies; the bottom half was not used for this paper. ChAdOx1-GFP and non-infected (N.I.) are also shown on the whole blots and were used as negative controls. From left to right: molecular weight, prME, prMEΔTM, E, EΔTM, GFP, non-infected cells.

## Data Availability

The data presented in this study is available in this article.

## Abbreviations

The following abbreviations are used in this manuscript:

ACK	Ammonium–Chloride–Potassium
ANOVA	Analysis of variance
AP	Alkaline phosphatase
AU	Arbitrary units
AWERB	Animal Welfare and Ethical Review Body
BAC	Bacterial artificial chromosome
ChAdOx1	Chimpanzee Adenovirus Oxford 1
C-term	Carboxyl-terminal
DBP	DNA-binding protein
DMSO	Dimethyl sulfoxide
E	Envelope
ELISA	Enzyme-linked immunosorbent assay
ELISpot	Enzyme-linked immuno-spot assay
ER	Endoplasmic reticulum
FBS	Fetal bovine serum
FELASA	Federation of European Laboratory Animal Science Associations
GFP	Green fluorescence protein
HAdV	Human adenovirus
ic	Intracranial
IFNAR	Interferon-α/β receptor
IFNγ	Gamma interferon
IgG	Immunoglobulin G
IM	Intramuscular
IP	Intraperitoneal
IP-10	Interferon gamma-induced protein-10
IU	Infectious units
LOD	Limit of detection
MOI	Multiplicity of infection
µl	Microliters
µm	Micrometers
nAb	Neutralizing antibodies
OAS-1	2′-5′-Oligoadenylate synthase 1
OD	Optical density
PBS	Phosphate-buffered saline
PFU	Plaque-forming units
PNGase F	Peptide N-glycosidase F
prM	Pre-membrane
PRNTs	Plaque Reduction Neutralization Test
PVDF	Polyvinylidene fluoride
ROI	Region of interest
RT	Room temperature
RT-qPCR	Reverse transcription–quantitative polymerase chain reaction
SD	Standard deviation
SFU	Spot-forming units
SNT	Serum neutralization test
TBS	Tris-buffered saline
TM	Transmembrane
tPA	Tissue plasminogen activator
WHO	World Health Organization
YFV	Yellow fever virus

## Appendix A

**Table A1:** Density readings of the bands and intensity ratios between YFV-E blots and DBP blots. The density readings were carried out using ImageJ.

	Density Readings			Intensity Ratios	
	Blot 1	Blot 2	Blot 3	Blot1/Blot3	Blot 2/Blot 3
prME	655,657.61	507,260.57	378,297.95	1.73	1.34
prMEΔTM	506,597.61	1,252,899.57	369,660.95	1.37	3.39
E	570,662.61	87,545.57	346,532.95	1.65	0.25
EΔTM	409,677.61	575,466.57	372,377.95	1.10	1.55
